# Systematic review of gut microbiota structural characteristics in patients with ischemic stroke

**DOI:** 10.3389/fcimb.2026.1884681

**Published:** 2026-07-21

**Authors:** Rong Tang, Ying Xu, Yu Zhou, Dixiao Yang, Qin Fu, Kunqin Xu, Lanhui Shi, Liangnan Zeng

**Affiliations:** 1 Anorectal Department, Hospital of Chengdu University of Traditional Chinese Medicine, Chengdu, Sichuan, China; 2 Department of Nursing, The Third People’s Hospital of Wenjiang District, Chengdu, Sichuan, China; 3 Department of Operating Theatre, Chengdu Fifth People’s Hospital, Chengdu, Sichuan, China; 4 Department of Nursing, Chengdu Fifth People’s Hospital, Chengdu, Sichuan, China; 5 Department of Emergency Medicine, Chengdu Fifth People’s Hospital, Chengdu, Sichuan, China; 6 Department of Nursing, Hospital of Chengdu University of Traditional Chinese Medicine, Chengdu, Sichuan, China

**Keywords:** dysbiosis, fecal microbiota, gut microbiota, ischemic stroke, systematic review

## Abstract

**Background:**

Emerging evidence implicates the gut microbiota (GM) in the pathogenesis of ischemic stroke (IS). However, systematic evaluations synthesizing evidence on microbial shifts, functional alterations, and clinical correlations are lacking. This study aimed to comprehensively analyze GM characteristics in IS patients versus healthy controls (HC).

**Methods:**

A systematic search of PubMed, Web of Science, and Embase was conducted from inception to April 2025. Observational studies comparing GM in IS patients and HC were included. Data on alpha/beta diversity, relative microbial abundance (phylum to species), predicted functional pathways, and microbiota–clinical indicator correlations were extracted. A narrative synthesis summarized the direction and consistency of reported findings.

**Results:**

Twenty-four studies involving 1,957 participants (1,159 IS, 798 HC) were included. The qualitative synthesis revealed no consistent differences in alpha diversity indices between IS and HC, while significant beta diversity separation was frequently reported (18/22 studies). At the phylum level, an increased relative abundance of Proteobacteria and a decreased abundance of Firmicutes were the most consistent findings in IS patients. Key genera frequently enriched in IS included *Lactobacillus*, *Streptococcus*, and *Parabacteroides*, while *Faecalibacterium*, *Agathobacter*, and *Blautia* were commonly depleted. LEfSe analysis confirmed these discriminative taxa. Predicted functional analysis suggested enrichment of pro-inflammatory pathways (e.g., lipopolysaccharide biosynthesis) and depletion of metabolic and neuroprotective pathways in IS. Clinically, *Faecalibacterium* abundance correlated negatively with NIHSS and mRS scores, while *Lactobacillus* and *Streptococcus* showed positive correlations with stroke severity and inflammatory markers.

**Conclusion:**

Ischemic stroke is associated with distinct gut microbial dysbiosis characterized by a pro-inflammatory, opportunistic pathogen-enriched, and butyrate-producer-depleted profile. These alterations correlate with disease severity and are linked to predicted disruptions in microbial metabolic functions. However, whether this dysbiosis is a cause, consequence, or exacerbating factor of IS remains to be determined. The gut microbiota may serve as a potential biomarker and therapeutic target in ischemic stroke.

**Systematic Review Registration:**

https://www.crd.york.ac.uk/PROSPERO/view/CRD420251117510, identifier CRD420251117510.

## 1 Introduction

Ischemic stroke (IS) remains a leading cause of mortality and long-term disability worldwide, with pathogenesis involving complex interactions between vascular, immune, and inflammatory pathways (Chen et al., 2025; Lian et al., 2025). Recently, the role of the gut–brain axis—a bidirectional communication network linking the central nervous system with the gastrointestinal tract—has gained considerable attention in cerebrovascular diseases (Lee et al., 2021; Guido et al., 2022; Li et al., 2025). The gut microbiota (GM), a diverse ecosystem of trillions of microorganisms, is a pivotal component of this axis, influencing host metabolism, immune regulation, and systemic inflammation (Pasokh et al., 2022; Pala et al., 2024).

Accumulating preclinical evidence suggests that GM dysbiosis can exacerbate cerebral ischemic injury by modulating immune responses, increasing intestinal permeability, and promoting the production of pro-atherogenic metabolites such as trimethylamine N-oxide (TMAO) (Sharma et al., 2021; Zang et al., 2023; Zhang and Yao, 2023). In humans, several case–control studies have reported altered GM composition in IS patients compared to healthy controls (HC), yet findings are often inconsistent regarding the direction of change for specific taxa and diversity indices (Zhang et al., 2024; Li et al., 2025). Furthermore, while individual studies have begun to explore functional metabolic shifts and correlations with clinical outcomes, a synthesized overview is lacking.

The recent systematic reviews have demonstrated the utility of meta-analysis in clarifying GM associations in related conditions. Building on this approach, we conducted a comprehensive systematic review and integrated analysis of existing studies to 1) quantify differences in GM alpha and beta diversity between IS patients and HC, 2) identify consistently altered microbial taxa from phylum to species level, 3) summarize predicted functional alterations in the GM metagenome, and 4) evaluate correlations between specific microbes and clinical indicators of stroke severity. By providing a holistic evidence synthesis, this work aims to establish a clearer picture of the “stroke microbiome” and its potential clinical implications.

Several systematic reviews have examined GM alterations in IS, primarily focusing on taxonomic composition (Lee et al., 2021; Zhang et al., 2024). However, none have systematically integrated functional predictions or clinical correlations across studies. The present review extends the current evidence base in four specific ways: 1) the largest taxonomic synthesis to date (24 studies, 1,957 participants) with vote-counting across all taxonomic levels, 2) the first systematic synthesis of predicted functional metagenomic alterations (PICRUSt/KEGG pathways) across studies, 3) a structured catalog of correlations between specific microbial taxa and clinical indicators (NIHSS, mRS, laboratory parameters), and 4) a critical examination of methodological heterogeneity and causal inference limitations. By integrating taxonomic, functional, and clinical correlation data, this synthesis aims to provide a holistic understanding of the GM’s role in IS and to identify the most robust associations for future mechanistic investigation.

## 2 Methods

### 2.1 Literature search

This systematic review was performed according to the guidance of the Preferred Reporting Items for Systematic Reviews and Meta-Analyses (PRISMA). The protocol was *a priori* registered in PROSPERO under registration number CRD420251117510. A systematic literature search was independently conducted by two investigators in both international and Chinese databases: PubMed, EMBASE, and Web of Science from their inception date until 2 April 2025, using the following search terms: (stroke OR ischemic stroke OR cerebral infarction OR acute ischemic stroke OR brain ischemia OR ischemic infarction OR cerebrovascular disease OR cerebrovascular accident) AND (gut OR gastrointestinal OR intestinal OR fecal OR fecal OR stool microbiome OR microbiota OR ecosystem OR bacteria OR flora OR microflora OR dysbiosis).

### 2.2 Study selection and data extraction

Cross-sectional, case–control, and cohort studies that fulfilled the following criteria were included: 1) intestinal microbiota studies comparing ischemic stroke patients with healthy controls, 2) GM samples derived from stool, and 3) publication in English. The exclusion criteria encompassed non-original studies, including reviews, conference papers, letters, and commentaries; non-human studies; non-English-language publications; and studies that did not assess alpha diversity or relative abundance in stroke and non-stroke groups.

The study selection was independently conducted by the same two investigators, and any disagreements were resolved by consulting a senior investigator. The two investigators reviewed the titles and abstracts first to identify relevant articles and then read the full texts for eligibility. If more than one paper was published based on the same study, only the paper with the largest sample size was included for analysis.

### 2.3 Data extraction

Data extraction was independently conducted by the same two investigators, and any disagreements were resolved by consulting a senior investigator. Data extraction was performed using a predetermined Excel sheet, and the following demographic and clinical characteristics were extracted: the first author, year of publication, the nation, study design, sample size, stroke subtype, microbiota species, detection method, and richness and diversity indices from 16S rRNA sequencing data, including the relative abundance of GM.

The primary outcomes were GM diversity (including alpha and beta diversity) and differences in GM abundance between patients with ischemic stroke and healthy controls. The primary and secondary outcomes were classified into three categories: significantly increased in ischemic stroke patients, significantly decreased in ischemic stroke patients, and no significant difference between ischemic stroke patients and controls.

### 2.4 Quality evaluation

Study quality was assessed using the Newcastle-Ottawa Scale (NOS) (Wells et al., 2000) for case–control or cohort studies and a modified AHRQ checklist for cross-sectional studies (Yang et al., 2019). The NOS evaluates the selection of study groups and comparability of groups in case–control and cohort studies, as well as the ascertainment of the exposure of interest. With a total score of 9, studies were rated as low quality (0–5) or high quality (6–9); low-quality studies were excluded.

### 2.5 Statistical analyses

Due to significant methodological heterogeneity (sequencing regions, bioinformatics pipelines), a formal quantitative meta-analysis of all effect sizes was not feasible for all outcomes. Instead, a semiquantitative synthesis approach was adopted.

For alpha diversity indices (Chao1, Shannon, Simpson, ACE), reported means/medians and significance values (*P*) for IS vs. HC from each study were tabulated. A narrative synthesis described the overall direction and consistency.

For microbial abundance, a vote-counting synthesis was performed based on the direction of change (increase/decrease in IS) and statistical significance (*P* < 0.05) reported in each study. The following rules were applied: A taxon was classified as “consistently increased” in IS if it was reported as significantly increased (*P* < 0.05) in at least two independent studies and no study reported a significant decrease in IS for that same taxon. A taxon was classified as “consistently decreased” in IS if it was reported as significantly decreased (*P* < 0.05) in at least two independent studies and no study reported a significant increase in IS for that same taxon. Taxa with conflicting reports (i.e., significant increase in one study and significant decrease in another) were classified as “inconsistent.” Taxa reported in only one study were listed individually in **Table 1** with their reported direction of change, but were not assigned a consistency classification as the definition requires at least two independent studies. For the purposes of this synthesis, study sample size and quality score were not used as weighting factors, as the number of studies per taxon was typically small (2–6); instead, the absolute number of supporting studies (*n*) was reported to allow readers to assess the strength of each finding. The full dataset, including the direction from each individual study, is available in **Table 1** for transparency. For phylum-level data reported consistently across >3 studies, we calculated pooled Hedges’ g with 95% confidence intervals (CI) using the Comprehensive Meta-Analysis Software (v3.3), employing a random-effects model. Heterogeneity was assessed with *I*^2^.

**Table 1:** Differences in relative abundance of gut microbiota between patients with ischemic stroke and healthy controls.

Taxonomic level	Microbial community name	Direction of change (case vs. control)	Reporting studies, n	Evidence summary (study reference)
Phylum	Bacteroidota	↑↓ (inconsistent)	7	(Cao et al., 2025) (↑), (Yu et al., 2024) (↓), (Ma et al., 2024) (↓), (Marsiglia et al., 2023) (↑), (Xu et al., 2021) (↓), (Li et al., 2020) (↓), (Xiang et al., 2020) (↑)
	Firmicutes	↓	6	(Ma et al., 2024) (↓), (Marsiglia et al., 2023) (↓), (Cheng et al., 2023) (↓), (Xu et al., 2021) (↑), (Xiang et al., 2020) (↓), (Li et al., 2020) (↓)
	Proteobacteria	↑	6	(Yu et al., 2024) (↑), (Chen et al., 2024) (↑), (Marsiglia et al., 2023) (↑), (Xu et al., 2021) (↑), (Xiang et al., 2020) (↑), (Li et al., 2020) (↑)
	Actinobacteria	↑	6	(Bao et al., 2024) (↑), (Marsiglia et al., 2023) (↓), (Jiang et al., 2023) (↑), (Xu et al., 2021) (↑), (Xiang et al., 2020) (LI vs. control:↓, AI vs. control: ↑), (Li et al., 2020) (↑)
	Verrucomicrobiota	↑	4	(Bao et al., 2024) (↑), (Marsiglia et al., 2023) (↑), 2022 (Zhang et al., 2022) (↑), (Xiang et al., 2020) (↑)
	Synergistetes	↑	3	(Huang et al., 2023) (↑), (Zhang et al., 2022) (↑), (Xiang et al., 2020) (↑)
	Fusobacteria	↑	2	(Chen et al., 2024) (↑), (Xiang et al., 2020) (↑)
	Cyanobacteria	↑	2	(Xiang et al., 2020) (↑), (Bao et al., 2024) (NS)
Class	Bacteroidia	↓	3	(Bao et al., 2024) (↑), (Xu et al., 2021) (↓), (Wang et al., 2018) (↓)
	Gammaproteobacteria	↑	2	(Xu et al., 2021) (↑), (Wang et al., 2018) (↑)
	Bacilli	↑	2	(Bao et al., 2024) (↑), (Xu et al., 2021) (↑)
	Clostridia	↑↓ (Inconsistent)	2	(Bao et al., 2024) (↓), (Xu et al., 2021) (↑)
	Deltaproteobacteria	↑↓ (Inconsistent)	2	(Bao et al., 2024) (↓), (Xu et al., 2021) (↑)
	Alphaproteobacteria	↑	1	(Bao et al., 2024) (↑)
Order	Lactobacillales	↑	2	(Bao et al., 2024) (↑), (Xu et al., 2021) (↑)
	Bacteroidales	↓	2	(Bao et al., 2024) (↓), (Xu et al., 2021) (↓)
	Enterobacterales	↑	1	(Xu et al., 2021) (↑)
	Lachnospirales	↓	1	(Bao et al., 2024) (↓)
Family	Streptococcaceae	↑	5	(Yu et al., 2024) (↑), (Ma et al., 2024) (↑), (Bao et al., 2024) (↑), (Marsiglia et al., 2023) (↑), (Zhang et al., 2022) (↑)
	Lactobacillaceae	↑	5	(Yu et al., 2024) (↓), (Ma et al., 2024) (↑), (Zhang et al., 2022) (↑), (Xu et al., 2021) (↑), (Xiang et al., 2020) (↑)
	Enterobacteriaceae	↑	5	(Marsiglia et al., 2023) (↑), (Jiang et al., 2023) (↑), (Zhang et al., 2022) (↑), (Xu et al., 2021) (↑), (Xiang et al., 2020) (↑)
	Lachnospiraceae	↓	4	(Bao et al., 2024) (↓), (Cheng et al., 2023) (↓), (Zhang et al., 2022) (↓), (Xiang et al., 2020) (↓)
	Ruminococcaceae	↓	4	(Marsiglia et al., 2023) (↓), (Zhang et al., 2022) (↓), (Xu et al., 2021) (↑), (Xiang et al., 2020) (↓)
	Bacteroidaceae	↑↓ (Inconsistent)	3	(Bao et al., 2024) (↓), (Marsiglia et al., 2023) (↑), (Xiang et al., 2020) (↑)
	Prevotellaceae	↑↓ (Inconsistent)	3	(Bao et al., 2024) (↓), (Cheng et al., 2023) (↑), (Xiang et al., 2020) (↓)
	Coriobacteriaceae	↓	2	(Marsiglia et al., 2023) (↓), (Xiang et al., 2020) (LI VS Control:↓, AI VS Control: ↑)
	Verrucomicrobiaceae	↑	2	(Xiang et al., 2020) (↑), (Marsiglia et al., 2023) (↑)
	Veillonellaceae	↑	1	(Xiang et al., 2020) (↑)
	Butyricicoccaceae	↓	1	(Yu et al., 2024) (↓)
	Clostridiaceae	↓	1	(Marsiglia et al., 2023) (↓)
	Bifidobacteriaceae	↓	1	(Xiang et al., 2020) (↓)
	Enterococcaceae	↓	1	(Xiang et al., 2020) (↓)
Genus	Lactobacillus	↑	6	(Yu et al., 2024) (↑), (Ma et al., 2024) (↑), (Xu et al., 2021) (↑), (Huang et al., 2021) (↑), (Xiang et al., 2020)(↑), (Li et al., 2020) (↑)
	Streptococcus	↑	4	(Yu et al., 2024) (↑), (Ma et al., 2024) (↑), (Lou et al., 2024) (↑), (Bao et al., 2024) (↑)
	Parabacteroides	↑	4	(Luo et al., 2024) (NIHSS > 5 subgroup: ↑), (Lou et al., 2024) (↑), (Jiang et al., 2023) (↑), (Xu et al., 2021) (↑)
	Faecalibacterium	↓	4	(Zhu et al., 2024) (↓), (Luo et al., 2024) (NIHSS > 5 subgroup: ↓), (Jiang et al., 2023) (↓), (Li et al., 2020) (↓)
	Prevotella	↓	4	(Bao et al., 2024) (↓), (Jiang et al., 2023) (↓), (Cheng et al., 2023) (↑), (Xiang et al., 2020) (↓)
	Agathobacter	↓	4	(Ma et al., 2024) (↓), (Luo et al., 2024) (NIHSS > 5 subgroup: ↓), (Bao et al., 2024) (↓), (Zhang et al., 2022) (↓)
	Eubacterium	↓	4	(Zhu et al., 2024) (↓), (Cheng et al., 2023) (↓), Xu 2021 (Xu et al., 2021) (↑), (Li et al., 2020) (↓)
	Blautia	↓	3	(Luo et al., 2024) (NIHSS > 5 subgroup: ↓), (Lou et al., 2024) (↓), (He et al., 2024) (↓)
	Christensenellaceae	↑	3	(Luo et al., 2024) (NIHSS > 5 subgroup: ↑), (Wei et al., 2023) (↑), (Marsiglia et al., 2023) (↑)
	Enterococcus	↑	3	(Wei et al., 2023) (↑), (Xu et al., 2021) (↑), (Huang et al., 2021) (↑)
	Klebsiella	↑	3	(Zhu et al., 2024) (↑), (Wei et al., 2023) (↑), (Xu et al., 2021) (↑)
	Bacteroides	↑↓ (Inconsistent)	3	(Bao et al., 2024) (↓), (Marsiglia et al., 2023) (↑), (Jiang et al., 2023) (↑)
	Akkermansia	↑	3	(Luo et al., 2024) (NIHSS > 5 subgroup: ↑), (Wei et al., 2023) (↑), (Marsiglia et al., 2023) (↑)
	Bifidobacterium	↑	3	(He et al., 2024) (↑), (Cheng et al., 2023) (↓), (Xu et al., 2021) (↑)
	Escherichia	↑↓ (Inconsistent)	2	(Bao et al., 2024) (↓), (Jiang et al., 2023) (↑)
	Roseburia	↓	2	(Jiang et al., 2023) (↓), (Li et al., 2020) (↓)
	Subdoligranulum	↓	2	(Luo et al., 2024) (NIHSS > 5 subgroup: ↓), (Zhang et al., 2022) (↓)
	Desulfovibrio	↑	2	(Wei et al., 2023) (↑), (Xu et al., 2021) (↑)
	Desulfovibrio	↑	2	(Wei et al., 2023) (↑), (Xu et al., 2021) (↑)
	Butyricicoccus	↓	2	(Wei et al., 2023) (↓), (Li et al., 2020) (↓)
	Butyricicoccus	↓	2	(Wei et al., 2023) (↓), (Li et al., 2020) (↓)
	Lachnoclostridium	↓	2	(Lou et al., 2024) (↓), (Li et al., 2020) (↓)
	Butyricimonas	↑	2	(He et al., 2024) (↑), (Wei et al., 2023) (↑)
	Lachnospira	↓	2	(Jiang et al., 2023) (↓), (Cheng et al., 2023) (↓)
	Lachnospira	↓	2	(Jiang et al., 2023) (↓), (Cheng et al., 2023) (↓)
	Ruminococcus	↓	1	(Xiang et al., 2020) (↓)
	Alistipes	↑	1	(Marsiglia et al., 2023) (↑)
	Anaerostipes	↓	1	(Marsiglia et al., 2023) (↓)
	Sutterella	↑	1	(Marsiglia et al., 2023) (↑)
	Fusobacterium	↑	1	(Zhu et al., 2024) (↑)
	Romboutsia	↓	1	(Zhu et al., 2024) (↓)
	Acetivibrio	↑	1	(Zhu et al., 2024) (↑)
	Dorea	↓	1	(He et al., 2024) (↓)
	Fusicatenibacter	↓	1	(Bao et al., 2024) (↓)
	Corynebacterium	↑	1	(Wei et al., 2023) (↑)
	Oscillospira	↑	1	(Wei et al., 2023) (↑)
	Phascolarctobacterium	↑	1	(Marsiglia et al., 2023) (↑)
	Shigella	↑	1	(Jiang et al., 2023) (↑)
	Methanobrevibacter	↑	1	(Cheng et al., 2023) (↑)
	Megasphaera	↑	1	(Zhang et al., 2022) (↑)
	Cloacibacillus	↑	1	(Zhang et al., 2022) (↑)
	Bilophila	↑	1	(Xu et al., 2021) (↑)
	Anaerotruncus	↑	1	(Xu et al., 2021) (↑)
	Hydrogenoanaerobacterium	↑	1	(Xu et al., 2021) (↑)
	Coprobacillus	↑	1	(Xu et al., 2021) (↑)
	Holdemania	↑	1	(Xu et al., 2021) (↑)
	Lactococcus	↑	1	(Li et al., 2020) (↑)
Species	Faecalibacterium prausnitzii	↓	2	(Luo et al., 2024) (NIHSS > 5 subgroup: ↓), (Cheng et al., 2023) (↓)
	*Bacteroides caccae*	↑	2	(Luo et al., 2024) (NIHSS > 5 subgroup: ↑), (Cheng et al., 2023) (↑)
	Bacteroides dorei	↑	1	(Luo et al., 2024) (NIHSS > 5 subgroup: ↑)
	Akkermansia muciniphila	↑	1	(Luo et al., 2024) (NIHSS > 5 subgroup: ↑)
	Butyricicoccus	↓	1	(Wei et al., 2023) (↓)
	Prevotella fusca	↑	1	(Cheng et al., 2023) (↑)
	Bacteroides thetaiotaomicron	↑	1	(Cheng et al., 2023) (↑)
	Bifidobacterium aesculapii	↓	1	(Cheng et al., 2023) (↓)
	Anaerostipes hadrus	↓	1	(Cheng et al., 2023) (↓)
	Blautia obeum	↓	1	(Huang et al., 2019) (↓)
	Streptococcus infantis	↑	1	(Huang et al., 2019) (↑)
	Prevotella copri	↑	1	(Huang et al., 2019) (↑)

↑ indicates a significant increase in the abundance of this bacterial community in the case group. ↓ indicates a significant decrease in the abundance of this bacterial community in the case group.

For beta diversity, functional pathways, and correlations, findings were summarized narratively and in structured tables, indicating the number of supporting studies and consistency of results.

Formal assessment of publication bias via funnel plots or Egger’s test was not performed because the number of studies included in each quantitative synthesis (*k* < 10) was insufficient to achieve adequate statistical power to distinguish chance from true asymmetry. For outcomes synthesized narratively or by vote counting, no validated method for assessing publication bias is currently available.

## 3 Results

### 3.1 Study characteristics and quality assessment

**Figure 1:**
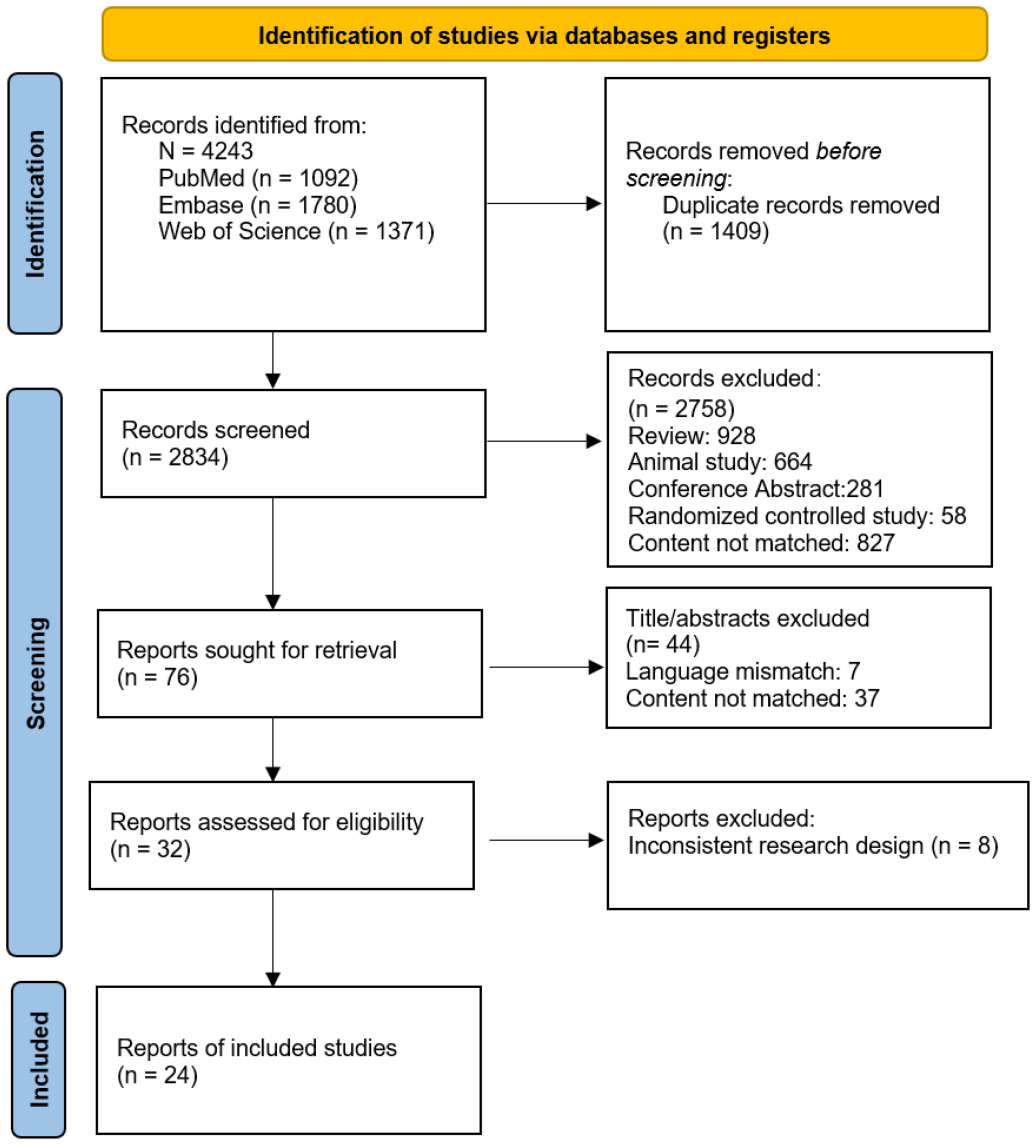
Flowchart of the study selection.

The flowchart of the study selection is shown in **Figure 1**.** A** total of 24 articles were included. Studies were mainly from China, Italy, and Thailand and accounted for 1,957 samples (1,159 stroke patients and 798 controls). The investigations were all observational in nature, comprising 16 case–control studies (Wang et al., 2018; Huang et al., 2019; Li et al., 2019; Li et al., 2020; Xiang et al., 2020; Huang et al., 2021; Xu et al., 2021; Zhang et al., 2022; Cheng et al., 2023; Huang et al., 2023; Marsiglia et al., 2023; Wei et al., 2023; Chen et al., 2024; He et al., 2024; Lou et al., 2024; Cao et al., 2025), 7 cross-sectional studies (Li et al., 2022; Samuthpongtorn et al., 2023; Bao et al., 2024; Luo et al., 2024; Ma et al., 2024; Yu et al., 2024; Zhu et al., 2024), and 1 cohort study (Jiang et al., 2023). Most studies used 16S rRNA gene sequencing of the V3–V4 regions (*n* = 19) on Illumina platforms (MiSeq, NovaSeq, HiSeq). Quality assessment scores ranged from moderate to high quality across all included studies (NOS: 7–9; AHRQ: 9–11). Detailed characteristics are shown in **Table 2**.

**Table 2:** Characteristics of studies included in this systematic review.

Study ID	Country	Study design	Total number (case/control)	Age (case/control)	Gender (male) (case/control)	BMI (case/control)	Hypertension (case/control)	Diabetes (case/control)	Disease type	Sample collection	Method to determine microbiota	Sequencing platform	Taxonomic unit	Quality score (tool)
(Cao et al., 2025)	China	Case–control study	15/15	64.60 ± 12.69/56.33 ± 8.25	9/7	NR	NR	NR	Ischemic stroke (original or recurrent)	Stool	16S rRNA gene amplification and sequencing V3–V4 region	Illumina MiSeq PE250	OTU (similarity > =97%)	8 (NOS)
(Zhu et al., 2024)	China	Cross-sectional study	50/46	66.42 ± 6.92/65.15 ± 5.82	26/25	Range from 18 to 24	36/0	25/0	Cerebral infarction	Stool	16S rRNA gene sequencing	Illumina NovaSeq	NR	10 (AHRQ)
(Yu et al., 2024)	China	Cross-sectional study	235/75	68 (60.0, 76.0)/64 (56.5, 72.0)a	110/38	NR	178/41	72/15	Acute ischemic stroke	Stool	16S rRNA gene amplification and sequencing	Illumina MiSeq	ASV (amplicon sequence variants)	9 (AHRQ)
(Ma et al., 2024)	China	Cross-sectional study	65/65	62.18 ± 11.19/60.74 ± 10.20	46/37	NR	58/32	24/19	Lacunar cerebral infarction	Stool	16S rRNA gene amplification and sequencing V3–V4 region	Illumina MiSeq	ASV (Amplicon sequence variants)	9 (AHRQ)
(Luo et al., 2024)	China	Cross-sectional study	59/31	67.66 ± 8.94/67.13 ± 6.30	42/17	NR	40/18	19/11	Acute ischemic stroke (anterior circulation)	Stool	16S rRNA gene amplification and sequencing V3–V4 region	Illumina NovaSeq6000	OTU (consistency > =97% )	9 (AHRQ)
(Lou et al., 2024)	China	Case–control study	48/10	65.92 ± 10.29/53.00 ± 4.00	33/5	22.94 (21.17, 26.38)/24.23 (21.40, 25.35)	NR	NR	Acute ischemic stroke	Stool	16S rRNA gene amplification and sequencing V4 region	Illumina NovaSeq	OTU (similarity > =97% )	8 (NOS)
(He et al., 2024)	China	Case–control study	63/36	66.25 ± 10.96/66.75 ± 5.86	43/20	24.67 ± 2.53/24.04 ± 2.97	42/15	23/6	Acute ischemic stroke (large vessel occlusion)	Stool	16S rRNA gene amplification and sequencing V3–V4 region	Illumina NovaSeq 6000	OTU (similarity 95% )	9 (NOS)
(Chen et al., 2024)	China	Case–control study	20/35	57.23 ± 8.61/59.41 ± 8.66	20/35	23.18 ± 3.09/23.07 ± 2.98	NR	NR	Ischemic stroke	Stool	16S rRNA gene amplification and sequencing V3–V4 region	Illumina HiSeq 2500	OTU (similarity 97% )	8 (NOS)
(Bao et al., 2024)	China	Cross-sectional study	47/15 (severe 10/moderate 21/mild 16/control 15)	Severe: 60.20 ± 14.48/moderate: 62.05 ± 13.46/mild: 66.50 ± 13.85/NC: 59.80 ± 6.14	6/15/8/9	NR	5/17/13/0	2/9/5/0	Ischemic stroke (acute and subacute)	Stool	16S rRNA gene amplification and sequencing V3–V4 region	Illumina NovaSeq PE250	ASV (amplicon sequence variants)	9 (AHRQ)
(Wei et al., 2023)	China	Case–control study	27/19	62.19 ± 8.75/59.58 ± 7.00	13/10	NR	24/8	10/3	Ischemic stroke	Stool	16S rRNA gene amplification and sequencing V3–V4 region	Illumina MiSeq	ASV (amplicon sequence variants)	8 (NOS)
(Samuthpongtorn et al., 2023)	Thailand	Cross-sectional study	14/15	61.1 ± 7.1/59.2 ± 8.2	12/12	NR	5/4	1/2	Ischemic stroke (large artery atherosclerosis)	Stool	16S rRNA gene amplification and sequencing V3–V4 region	Illumina MiSeq	OTU	9 (AHRQ)
(Marsiglia et al., 2023)	Italy	Case–control study (Including longitudinal follow-up: T0 = early stage of rehabilitation, T1 = 6 weeks later)	10/21 (metabolic analysis subset: 10/6)	70.5 (14.0)/66.0 (9.0)	6/NR	27.36 ± 5.32/NR	8/NR	2/NR	Ischemic stroke (rehabilitation period)	Stool	16S rRNA gene amplification and sequencing V3–V4 region	Illumina	ASV	9 (NOS)
(Jiang et al., 2023)	China	Prospective observational study	90/60 (severe 16, mild–moderate 74)	59.77 ± 11.36/60.75 ± 8.72	60/41	24.63 ± 2.96/24.14 ± 1.96	81/14	61/2	Acute ischemic stroke	Stool	16S rRNA gene amplification and sequencing V3–V4 region	Illumina HiSeq 2500	OTU (closed-reference)	9 (NOS)
(Huang et al., 2023)	China	Case–control study	20/20 (fecal samples 17/18)	65.0 ± 11.17/63.60 ± 9.58	12/10	NR	9/NR	4/NR	Acute Ischemic Stroke (Phlegm-Heat Pattern)	Stool	16S rRNA gene amplification and sequencing V4 region	Illumina HiSeq 2500	OTU (similarity 97%)	8 (NOS)
(Cheng et al., 2023)	China	Retrospective case–control	CI: 12/CI-T2DM: 12/control: 12	CI: 64.42 ± 3.15/CI-T2DM: 64.75 ± 2.83/Control: 61.92 ± 2.97	CI: 6/CI-T2DM: 6/control: 6	NR	0/0/0	0/CI-T2DM: 12 (10.83 ± 2.31 years)/0	Cerebral infarction (CI)	Stool	Metagenomic sequencing	Illumina NovaSeq	OTU	8(NOS)
(Zhang et al., 2022)	China	Case–control study	82/82	62.57 ± 9.26/63.38 ± 9.34	49/49	25.58 ± 3.84/24.47 ± 3.33	NR	NR	Ischemic stroke	Stool	16S rRNA gene sequencing V4 region	Ion S5™ XL	OTU (similarity 97%)	9 (NOS)
(Li et al., 2022)	China	Cross-sectional study	tAIS: 7/ntAIS: 10/control: 10	tAIS: 71.57 ± 9.95/ntAIS: 67.50 ± 13.79/HC: 63.30 ± 7.29	tAIS: 4/ntAIS: 5/control: 5	tAIS: 23.43 ± 1.16/ntAIS: 23.98 ± 1.23/HC: 22.59 ± 1.57	tAIS: 4/ntAIS: 6/HC: 3	tAIS: 3/ntAIS: 5/HC: 3	Acute ischemic stroke	Stool	16S rRNA gene amplification and sequencing V3–V4 region	Illumina	OTU (similarity > =97%)	10 (AHRQ)
(Xu et al., 2021)	China	Case–control study	LAA: 61/CE: 20/control: 51	>60 years: LAA 41/CE 17/control 22	LAA: 40/CE: 11/control: 19	NR	LAA: 42/CE: 16/control: 15	LAA: 24/CE: 3/control: 4	Ischemic stroke	Stool	16S rRNA gene amplification and sequencing V4–V5 region	Illumina MiSeq	OTU (identity 97%)	9 (NOS)
(Huang et al., 2021)	China	Case–control study	27/19	62.19 ± 9.71/NR	14/NR	NR	12/NR	4/NR	Ischemic stroke	Stool	16S rRNA gene amplification and sequencing V3–V4 region	Illumina MiSeq	OTU (similarity > =97%)	8 (NOS)
(Xiang et al., 2020)	China	Case–control study	30/16 (LI: 10, AI:10, PI: 10/control: 16)	LI:72 (57–89)/AI: 73 (59–87)/PI: 73 (60–83)/NC: 71 (61–81)	LI: 4/AI: 5/PI: 5/control: 7	LI: 24.8 (21.2–28.4)/AI: 25.1 (21.8–28.1)/PI: 24.9 (20.2–28.6)/control: 24.1 (20.4–27.2)	NR	NR	Acute ischemic stroke (lacunar infarction or non-lacunar acute ischemic infarction and post-ischemic stroke)	Stool	16S rRNA gene amplification and sequencing V3 region	Illumina MiSeq	OTU (similarity 97%)	8 (NOS)
(Li et al., 2020)	China	Case–control study	79/98	66.61 ± 12.07/64.01 ± 10.44	50/57	24.22 ± 2.34/23.83 ± 2.11	56/58	28/22	Cerebral infarction (CI)	Stool	16S rRNA gene amplification and sequencing V3–V4 region	Illumina MiSeq	OTU (similarity 97%)	9 (NOS)
(Li et al., 2019)	China	Case–control study	30/30	60.47 ± 10.57/64.17 ± 12.67	21/18	NR	NR	NR	Cerebral ischemic stroke (CI)	Stool	16S rRNA gene amplification and sequencing V1–V2 region	Illumina MiSeq	OTU (similarity 97%)	8 (NOS)
(Huang et al., 2019)	China	Case–control study	31/9	Median: 61 (40–94)/61 (53–69)a	22/6	NR	23/2	0/0	Acute cerebral infarction (large artery atherosclerotic subtype)	Stool	16S rRNA gene amplification and sequencing V4 region	Illumina MiSeq	OTU	7 (NOS)
(Wang et al., 2018)	China	Case–control study	10/10	Average: 64 years (range: 53–82)/same source, exact split NR	Total 10M/10F, exact group split NR	NR	NR	NR	Cerebral infarction (CI)	Stool	16S rRNA gene sequencing V4 region	Illumina HiSeq2500 PE250	OTU	7 (NOS)

NR, not reported; CI-T2DM, cerebral infarction with type 2 diabetes mellitus; tAIS, patients with PHS; ntAIS, patients with non-PHS; LI, lacunar infarction; AI, non-lacunar acute ischemic infarction; PI, post-ischemic stroke; LAA, large artery atherosclerotic stroke; CE, cardioembolic stroke; NOS, Newcastle-Ottawa Scale; AHRQ, modified Agency for Healthcare Research and Quality checklist.

^a^ Median (interquartile range).

### 3.2 Alpha and beta diversity

Of the 24 included studies, 21 reported results for at least one alpha diversity index. The findings were inconsistent. Among these, 11 studies (52.4%) reported at least one index with a statistically significant difference (*P* < 0.05) between IS patients and HC. However, the direction of change (increase or decrease in IS) varied across studies and across different indices. The remaining 10 studies reported no statistically significant differences for any of the reported alpha diversity indices. Meta-analysis was not performed due to high heterogeneity in reporting formats (means, medians, *P*-values only).

In contrast, analyses of beta diversity (reported in 22 studies) demonstrated a more consistent pattern. A significant separation in overall microbial community structure between IS and HC groups was reported in 18 studies (81.8%), as determined by ordination analyses (PCoA, NMDS, PCA) or statistical tests (ANOSIM, PERMANOVA) using distance metrics such as Bray–Curtis or UniFrac (*P* < 0.05). Three studies found no significant separation, and one did not provide a clear statistical conclusion (**Table 3**).

**Table 3:** Analysis of alpha diversity and beta diversity in gut microbiota between patients with ischemic stroke and healthy controls.

Study ID	α-Diversity				β-Diversity				
	Chao1 (case/control)	Shannon (case/control)	Simpson (case/control)	Ace (case/control)					
	p-value	p-value	p-value	p-value	Analysis method	Distance matrix	Statistics	p	Conclusion
(Cao et al., 2025)	p > 0.05	**p < 0.05**	**p < 0.05**	p > 0.05	ANOSIM	Weighted UniFrac/unweighted UniFrac	Weighted UniFrac R = 0.144, Unweighted UniFrac R = 0.117	Weighted UniFrac **p = 0.004**, unweighted UniFrac **p = 0.011**	Distinct separation
(Zhu et al., 2024)	p > 0.05	p > 0.05	p > 0.05	NR	NMDS	Weighted UniFrac/unweighted UniFrac	Weighted UniFrac stress= 0.15, unweighted UniFrac stress = 0.17	Weighted UniFrac **p < 0.05**, unweighted UniFrac **p < 0.05**	Distinct separation
(Yu et al., 2024)	NR	p > 0.05	p > 0.05	NR	PCoA	NR	NR	**p < 0.05**	Distinct separation
(Ma et al., 2024)	NR	**p < 0.05**	**p < 0.05**	NR	PCoA/ANOSIM	Hellinger/Bray–Curtis/weighted UniFrac	Hellinger, R2 = 0.0105, Bray–Curtis, R2 = 0.0146	Hellinger **p = 0.043**, Bray–Curtis **p = 0.055**	The authors reported significant differences (although p > 0.05)
(Luo et al., 2024)	p > 0.05	p > 0.05	p > 0.05	NR	PCoA	NR	NR	**p < 0.01**	Significant difference
(Lou et al., 2024)	p > 0.05	p > 0.05	p > 0.05	p > 0.05	PCoA	NR	NR	**p < 0.001**	Significant difference
(He et al., 2024)	**p < 0.05**	p > 0.05	p > 0.05	**p < 0.05**	PCoA	Weighted UniFrac	NR	NR	No clear qualitative conclusion was provided
(Chen et al., 2024)	p > 0.05	p > 0.05	**p < 0.05**	p > 0.05	PCA	Weighted UniFrac/unweighted UniFrac	NR	NR	NR
(Bao et al., 2024)	NR	**p < 0.05** case ↓	p > 0.05	NR	PCoA	Weighted UniFrac/unweighted UniFrac/Bray–Curtis/Jaccard	Weighted UniFrac F = 1.80, unweighted UniFrac F = 2.42, Bray–Curtis F = 1.70, Jaccard F = 1.69	Weighted UniFrac **p = 0.05**, unweighted UniFrac **p = 0.001**, Bray–Curtis **p = 0.001**, Jaccard **p = 0.001**	Significant difference (between the control group and stroke groups with different degrees of severity)
(Wei et al., 2023)	p > 0.05	p > 0.05	**p < 0.05**	p > 0.05	PCoA	Bray–Curtis	R = 0.233	**p = 0.001**	Significant difference (among the healthy control group, stroke group, and post-stroke epilepsy group)
(Samuthpongtorn et al., 2023)	NR	p > 0.05	NR	NR	PCoA	Bray–Curtis	NR	**Order level: p = 0.017, family level: p = 0.011, genus level: p = 0.003**	Significant difference
(Marsiglia et al., 2023)	**p < 0.05** Case ↓	p > 0.05	p > 0.05	NR	PCoA	Bray-Curtis	NR	**p = 0.001**	Distinct separation [IS patients (T0 and T1) versus healthy control group]
(Jiang et al., 2023)**	NR	NR	NR	NR	PCoA	NR	NR	NR	Distinct separation (mild–moderate group, severe group, and control group. Group separation increased with severity)
(Huang et al., 2023)	p > 0.05	p > 0.05	p > 0.05	NR	PCoA	NR	NR	NR	Distinct separation
(Cheng et al., 2023)	NR	NR	NR	NR	PCoA	Bray–Curtis	NR	NR	The result of PCoA analysis showed that there was a crossover among the three groups, as well as a different trend (cerebral infarction, CI-T2DM: cerebral infarction complicated with type 2 diabetes mellitus, healthy controls)
(Zhang et al., 2022)	NR	NR	NR	NR	PCoA/ANOSIM	Unweighted UniFrac	Unweighted UniFrac R = 0.067	**p = 0.001**	Significant difference (IS patients exhibit greater microbial diversity)
(Zhang et al., 2022)	p < 0.05	p > 0.05	p > 0.05	AIS vs. control p > 0.05	PCoA/ANOSIM	NR	R = 0.217	**p = 0.005**	Significant difference
(Xu et al., 2021)	NR	LAA vs. control: p < 0.05 case ↑ CE vs. control: p > 0.05	LAA vs. control: p < 0.05 case ↓	NR	PCoA/NMDS	Weighted/unweighted UniFrac/Bray–Curtis	LAA vs. control: R = 0.08	LAA vs. control: **p = 0.004**, CE vs. control: **p > 0.05**	Significant difference (LAA vs. control)
(Huang et al., 2021)	p > 0.05	p > 0.05	p > 0.05	p > 0.05	PCoA/PCA/NMDS	NR	NR	NR	NR
(Xiang et al., 2020)	**AI vs. control: p < 0.05**	**PI vs. control: p < 0.05**	**PI vs. control: p < 0.05**	**AI vs. control: p < 0.05**	PCoA/PCA/NMDS/ANOSIM	Bray–Curtis	ANOSIM R = 0.371	**p = 0.001**	PCA and PCoA indicated AI differed significantly from the LI and PI groups and was comparable to the NC group. ANOSIM differed significantly among the three groups. NMDS analysis based on the Bray–Curtis distances revealed significant differences between AI patients and the other three groups
(Li et al., 2020)	p > 0.05	p > 0.05	p > 0.05	p > 0.05	PCoA	NR	NR	NR	No significant difference
(Li et al., 2019)	p > 0.05	p > 0.05	p > 0.05	p > 0.05	PCoA/ANOSIM	Weighted UniFrac/unweighted UniFrac	ANOSIM: weighted UniFrac R = −0.012, unweighted UniFrac R = 0.0254	Weighted UniFrac **p = 0.132**, unweighted UniFrac **p = 0.649**	No significant difference
(Huang et al., 2019)	**p > 0.05**	p > 0.05	p > 0.05	p > 0.05	PCA/PLS-DA	Weighted UniFrac/unweighted UniFrac	NR	NR	No significant difference
(Wang et al., 2018)	p < 0.05 case↓	NR	NR	NR	PCoA/UPGMA/heatmap	Weighted UniFrac/unweighted UniFrac	NR	NR	Significant difference

LAA, large artery atherosclerotic stroke; IS, ischemic stroke; LI, lacunar infarction; AI, non-lacunar acute ischemic infarction; PI, post-ischemic stroke; ANOSIM, analysis of similarities; PCoA, principal coordinates analysis; PCA, principal component analysis; NMDS, non-metric multidimensional scaling; PLS-DA, partial least squares discrimination analysis; UPGMA, unweighted pair-group method with arithmetic mean analysis.

*Median (interquartile range), **mild–moderate vs. control: *p* < 0.05, severe vs. control: *p* < 0.01; bold *p*-values indicate statistical differences.

### 3.3 Alterations in microbial composition

Phylum level: Changes at the phylum level were synthesized from multiple studies. The most consistent finding was an increase in the relative abundance of Proteobacteria in IS patients, reported in six studies (Li et al., 2020; Xiang et al., 2020; Xu et al., 2021; Marsiglia et al., 2023; Chen et al., 2024; Yu et al., 2024). A decrease in Firmicutes was also a common finding, reported in five studies (Li et al., 2020; Xiang et al., 2020; Cheng et al., 2023; Marsiglia et al., 2023; Ma et al., 2024). Changes in Bacteroidota and Actinobacteria were inconsistent across studies, with reports of both increases and decreases (**Table 1**).

Genus level: The vote-counting synthesis identified genera with consistent directional changes. Key taxa significantly increased in IS patients included *Lactobacillus* (six studies), *Streptococcus* (four studies), and *Parabacteroides* (four studies). Taxa significantly decreased in IS included *Faecalibacterium* (four studies), *Agathobacter* (four studies), and *Blautia* (three studies) (**Table 1**). LEfSe analysis from 14 studies supported these findings, with genera such as *Escherichia_Shigella*, *Streptococcus*, and *Lactobacillus* frequently identified as discriminators for IS, while *Faecalibacterium* and members of the Lachnospiraceae family were often associated with HC (**Table 4**).

**Table 4:** Differential gut microbial communities associated with ischemic stroke based on LEfSe analysis (ranked by LDA effect values).

	Enriched in IS patients			Enriched in healthy controls		
Taxonomic level	LDA > =4.0	LDA 3-3.9	LDA 2-2.9	LDA > =4.0	LDA 3–3.9	LDA 2–2.9
Phylum	Proteobacteria (Li et al., 2022)	Bacteroidota (Luo et al., 2024)	Actinobacteria (Xu et al., 2021)	Firmicutes (Li et al., 2022; Cheng et al., 2023)	Actinobacteria (Cheng et al., 2023)	Euryarchaeota (Cheng et al., 2023)
	Verrucomicrobia (Li et al., 2022)	Proteobacteria (Xu et al., 2021; Chen et al., 2024)	Parcubacteria (Li et al., 2019)		Bacteroidetes (Xu et al., 2021)	
	Actinobacteria (Li et al., 2022; Cao et al., 2025)	Firmicutes (Xu et al., 2021)				
		Verrucomicrobia (Li et al., 2019)				
Class	Gammaproteobacteria (Li et al., 2022; Zhang et al., 2022)	Clostridia (Xu et al., 2021; Ma et al., 2024)	Bacilli (Xu et al., 2021; Ma et al., 2024; Zhu et al., 2024)	Clostridia (Li et al., 2022; Zhang et al., 2022; Cheng et al., 2023)	Clostridia (Luo et al., 2024)	Methanobacteria (Cheng et al., 2023)
	Bacilli (Zhang et al., 2022; Lou et al., 2024)	Bacteroidia (Luo et al., 2024)	Erysipelotrichia (Xu et al., 2021)		Actinobacteria (Cheng et al., 2023)	
	Gammaproteobacteria (Li et al., 2022)	Gammaproteobacteria (Xu et al., 2021; Chen et al., 2024)			Bacteroidia (Xu et al., 2021)	
	Verrucomicrobiae (Li et al., 2022)	Verrucomicrobiae (Li et al., 2019)				
	Actinobacteria (Li et al., 2022; Cao et al., 2025)					
Order	Enterobacterales (Li et al., 2022; Zhang et al., 2022)	Oscillospirales (Ma et al., 2024)	Xanthomonadales (He et al., 2024)	Clostridiales (Li et al., 2022; Cheng et al., 2023; Marsiglia et al., 2023; Cao et al., 2025)	Bacteroidales (Xu et al., 2021)	Rickettsiales (Lou et al., 2024)
	Lactobacillales (Zhang et al., 2022; Lou et al., 2024)	Bacteroidales (Luo et al., 2024)	Lactobacillales (Xu et al., 2021)	Lachnospirales (Zhang et al., 2022)	Lachnospirales (Luo et al., 2024)	Pseudomonadales (Cheng et al., 2023; He et al., 2024; Lou et al., 2024)
	Bifidobacteriales (Li et al., 2022; Cao et al., 2025)	Clostridiales (Xu et al., 2021)	Erysipelotrichales (Xu et al., 2021)		Erysipelotrichales (Lou et al., 2024)	Xanthomonadales (Lou et al., 2024)
	Verrucomicrobiales (Li et al., 2022)	Enterobacteriales (Xu et al., 2021; Chen et al., 2024)			Clostridiales (Lou et al., 2024)	Pasteurellales (He et al., 2024)
		Verrucomicrobiales (Li et al., 2019)			Bifidobacteriales (Cheng et al., 2023)	Lactobacillales (Xiang et al., 2020)
						Methanobacteriales (Cheng et al., 2023)
Family	Enterobacteriaceae (Li et al., 2022; Zhang et al., 2022; Marsiglia et al., 2023)	Tannerellaceae (Cheng et al., 2023; Lou et al., 2024)	Anaerovoracaceae (Zhu et al., 2024)	Ruminococcaceae (Zhang et al., 2022; Cao et al., 2025)	Ruminococcaceae (Luo et al., 2024)	Atopobiaceae (Zhu et al., 2024)
	Streptococcaceae (Zhang et al., 2022; Lou et al., 2024)	Lactobacilliaceae (He et al., 2024; Zhu et al., 2024)	Synergistaceae (Zhu et al., 2024)	Lachnospiraceae (Li et al., 2022; Zhang et al., 2022; Cheng et al., 2023; Lou et al., 2024)	Lachnospiraceae (Chen et al., 2024; Luo et al., 2024)	Oscillospiraceae (Zhu et al., 2024)
	Lactobacillaceae (Zhang et al., 2022; Ma et al., 2024)	Streptococcaceae (Ma et al., 2024; Zhu et al., 2024)	Bacillaceae (Xiang et al., 2020; Ma et al., 2024)	Prevotellaceae (Lou et al., 2024)	Clostridiaceae (Lou et al., 2024)	Lachnospiraceae (Ma et al., 2024)
	Tannerellaceae (Lou et al., 2024)	Bacteroidaceae (Luo et al., 2024)	Paenibacillaceae (Xiang et al., 2020)	Coriobacteriaceae (Marsiglia et al., 2023)	Bifidobaicteriaceae (Cheng et al., 2023)	Alcaligenaceae (Lou et al., 2024)
	Veillonellaceae (Lou et al., 2024)	Muribaculaceae (Lou et al., 2024)	Xanthomonadaceae (Xiang et al., 2020; He et al., 2024)		Eubacteriaceae (Cheng et al., 2023)	Comamonadaceae (Lou et al., 2024)
	Porphyromonadaceae (He et al., 2024)	Enterococcaceae (He et al., 2024)	Erythrobacteraceae (He et al., 2024)			Morganellaceae (Lou et al., 2024)
	Pseudomonadaceae (Marsiglia et al., 2023)	Enterobacteriaceae (Xu et al., 2021; Chen et al., 2024)	Lactobacillaceae (Xu et al., 2021)			Moraxellaceae (Lou et al., 2024)
	Akkermansiaceae (Li et al., 2022)	Erysipelotrichaceae (Marsiglia et al., 2023)	Erysipelotrichaceae (Xiang et al., 2020; Xu et al., 2021)			Pseudomonadaceae (Cheng et al., 2023; Lou et al., 2024)
	Bifidobacteriaceae (Li et al., 2022)	Rikenellaceae (Marsiglia et al., 2023)	Bacteroidaceae (Xiang et al., 2020)			Xanthomonadaceae (Lou et al., 2024)
	Christensenellaceae (Li et al., 2022)	Preyotellaceae (Cheng et al., 2023)	Weeksellaceae (Xiang et al., 2020)			Helicobacteraceae (Lou et al., 2024)
		Ruminococcaceae (Xu et al., 2021)	Brucellaceae (Xiang et al., 2020)			Pasteurellaceae (He et al., 2024)
		Porphyromonadaceae (Xu et al., 2021)	Methanobacteriaceae (Xiang et al., 2020)			Methanobaicteriaceae (Cheng et al., 2023)
		Verrucomicrobiaceae (Li et al., 2019)	Pseudomonadaceae (Xiang et al., 2020)			Lactobacillaceae (Xiang et al., 2020)
			Verrucomicrobiaceae (Xiang et al., 2020)			Clostridiaceae (Li et al., 2019)
			Sphingomonadaceae (Xiang et al., 2020)			
			Flavobacteriaceae (Li et al., 2019)			
Genus	Escherichia_Shigella (Li et al., 2022)	Bacteroides (Luo et al., 2024)	Desulfovibrio (Zhu et al., 2024)	Faecalibacterium (Li et al., 2022; Zhang et al., 2022; He et al., 2024; Cao et al., 2025)	Citrobacter (Zhu et al., 2024)	Haemophilus (He et al., 2024)
	Streptococcus (Zhang et al., 2022; Marsiglia et al., 2023)	Lactobacillus (He et al., 2024; Zhu et al., 2024)	Lactobacillus (Xu et al., 2021)	Agathobacter (Li et al., 2022)	Acidaminococcus (Zhu et al., 2024)	Fastidiosipila (Cheng et al., 2023)
	Lactobacillus (Zhang et al., 2022; Ma et al., 2024)	Streptococcus (Ma et al., 2024; Zhu et al., 2024)	Holdemanella (Zhu et al., 2024)	Methanobrevibacter (Marsiglia et al., 2023)	Agathobacter (Ma et al., 2024)	Dakarella (Cheng et al., 2023)
	Bacteroides (Marsiglia et al., 2023)	Veillonella (Ma et al., 2024)	Acidaminococcus (Ma et al., 2024)		Faecalibacterium (Luo et al., 2024)	Methanobrevibacter (Cheng et al., 2023)
	Akkermansia (Zhang et al., 2022; Marsiglia et al., 2023)	Enterococcus (He et al., 2024)	Bacillus (Ma et al., 2024)		Blautia (Luo et al., 2024)	Pyramidobacter (Cheng et al., 2023)
	Alistipes (Marsiglia et al., 2023)	Escherichia (Chen et al., 2024)	Peptoclostridium (Ma et al., 2024)		Anaerostipes (Cheng et al., 2023; He et al., 2024)	Pseudomonas (Cheng et al., 2023)
	Oscillospira (Marsiglia et al., 2023)	Parabacteroides (Xu et al., 2021; Cheng et al., 2023)	Intestinibacter (Ma et al., 2024)		Dorea (He et al., 2024)	Anaerostipes (Li et al., 2019)
	Christensenellaceae (Marsiglia et al., 2023)	Porphyromonas (Cheng et al., 2023)	Alloscardovia (Ma et al., 2024)		Butyricococcus (He et al., 2024)	
	Prevotella (Marsiglia et al., 2023)	Klebsiella (Xu et al., 2021)	Cloacibacillus (Ma et al., 2024)		Aestuarispira (He et al., 2024)	
	Sutterella (Marsiglia et al., 2023)	Odoribacter (Li et al., 2019)	Alloprevotella (Xu et al., 2021; Chen et al., 2024)		Catenibacterium (Marsiglia et al., 2023)	
	Bifidobacterium (Li et al., 2022; Marsiglia et al., 2023)	Akkermansia (Li et al., 2019)	Butyricimonas (Chen et al., 2024)		Anaerotruncus (Marsiglia et al., 2023)	
	Desulfovibrio (Marsiglia et al., 2023)		Fastidiosipila (Xu et al., 2021)		Erwinia (Cheng et al., 2023)	
	Parabacteroides (Marsiglia et al., 2023)		Anaerotruncus (Xu et al., 2021)		Bifidobacterium (Cheng et al., 2023)	
	Phascolarctobacterium (Marsiglia et al., 2023)		Mogibacterium (Xu et al., 2021)		Fusicatenibacter (Cheng et al., 2023)	
	Megasphaera (Marsiglia et al., 2023)		Collinsella (Xu et al., 2021)		Eubacterium (Cheng et al., 2023)	
	Clostridium (Marsiglia et al., 2023)		Victivallis (Li et al., 2019)		Roseburia (Cheng et al., 2023)	
	Butyricimonas (Marsiglia et al., 2023)					
	Pyramidobacter (Marsiglia et al., 2023)					
Species	Escherichia coli (Chen et al., 2024)	Escherichia_coli (Chen et al., 2024)	Campylobacter_hominis (Xu et al., 2021)		Faecalibacterium prausnitzii (Luo et al., 2024)	Roseburia_hominis (Chen et al., 2024)
		Bacteroides_coprocola (He et al., 2024)	Mogibacterium_diversum (Xu et al., 2021)			
			Synergistes_jonesii (Xu et al., 2021)			
			Prevotella stercorea (Xu et al., 2021)			
			Sutterella_parvirubra (Chen et al., 2024)			
			Porphyromonas_somerae (Xu et al., 2021)			

LDA scores are used to estimate the magnitude of each cluster’s contribution to intergroup differences, where LDA > =4.0 indicates a substantial contribution, LDA 3.0–4.0 denotes a moderate contribution, and LDA >2.0 signifies a minor contribution.

LDA, linear discriminant analysis.

### 3.4 Predicted functional alterations

Predicted functional changes in the gut microbiome of IS patients are summarized in **Table 5**. Eleven studies performed PICRUSt/KEGG analysis. IS patients showed enrichment in pathways related to bacterial proliferation (e.g., translation, pyrimidine metabolism) and potential virulence (e.g., lipopolysaccharide biosynthesis, flagellar assembly) (Huang et al., 2019; Marsiglia et al., 2023; Cao et al., 2025). Conversely, pathways involved in host-nutritive metabolism, including amino acid metabolism and metabolism of cofactors and vitamins, were frequently downregulated (Xu et al., 2021; Bao et al., 2024; Lou et al., 2024). A notable finding concerns butanoate metabolism. While key SCFA-producing genera were depleted, the genetic potential for this pathway was reported as enriched in the IS group in one study (Marsiglia et al., 2023). Additionally, other metabolic perturbations were observed, such as downregulation in oxidative phosphorylation and upregulation in membrane transport (Xu et al., 2021; Bao et al., 2024; Lou et al., 2024).

**Table 5:** Comparative analysis of predicted gut microbiota functional potentials between patients with ischemic stroke and healthy controls (based on PICRUSt/KEGG).

KEGG pathway/functional category	Alterations in the stroke group (case)	Alterations in the control group
Translation	Enriched (LDA ≥ 2.0) (Cao et al., 2025)	–
Aminoacyl-tRNA biosynthesis	–	Enriched (LDA 3–4) (Marsiglia et al., 2023)
Ribosome	–	Enriched (LDA 3–4) (Marsiglia et al., 2023)
Transcription machinery	↓ (Lou et al., 2024)	–
Amino acid metabolism	↓ (Level 2) (Xu et al., 2021)	↑ (Li et al., 2020)
	↑ (Cao et al., 2025)	
Histidine metabolism	↑ (Level 3) (Ma et al., 2024)	Enriched (LDA 3–4) (Marsiglia et al., 2023)
Tryptophan biosynthesis	↓ (Wei et al., 2023)	–
Pyrimidine metabolism	Enriched (LDA ≥ 2.0) (Cao et al., 2025)	Enriched (LDA 3–4) (Marsiglia et al., 2023)
	↓ (Level 3) (Lou et al., 2024)	–
Purine metabolism	–	Enriched (LDA 3–4) (Marsiglia et al., 2023)
Pyrimidine deoxyribonucleotide biosynthesis	↑ (Wei et al., 2023)	–
Oxidative phosphorylation	↓ (Level 3) (Lou et al., 2024)	–
Citrate cycle (TCA cycle)	↑ (He et al., 2024)	Highly enriched in both groups (Xiang et al., 2020)
Methane metabolism	↑ (Level 3) (Xu et al., 2021)	Enriched (LDA 3–4) (Marsiglia et al., 2023)
	↓ (Huang et al., 2019)	–
Pyruvate metabolism	–	Enriched (LDA ≥ 2.0) (Cao et al., 2025)
Membrane transport	↑ (Level 2) (Xu et al., 2021; Bao et al., 2024)	–
Two-component system	–	Enriched (LDA ≥ 2.0) (Cao et al., 2025)
Signal transduction	↑ (Level 2) (Li et al., 2020; Xu et al., 2021)	Enriched (LDA ≥ 2.0) (Cao et al., 2025)
Lipopolysaccharide biosynthesis	Enriched (LDA 3–4; ↑ (Huang et al., 2019; Marsiglia et al., 2023)	–
Flagellar assembly	Enriched (LDA 3–4); ↑ (Huang et al., 2019; Marsiglia et al., 2023)	–
Metabolism of cofactors and vitamins	↓ (Level 2) (Xu et al., 2021; Bao et al., 2024; Lou et al., 2024)	–
Riboflavin metabolism	↓ (Level 3) (Lou et al., 2024)	–
Thiamine metabolism	↑ (Level 3) (Ma et al., 2024)	–
Xenobiotics biodegradation and metabolism	↑ (Level 2) (Xu et al., 2021; Bao et al., 2024)	–
Xylene degradation	↓ (He et al., 2024)	–
Glutathione metabolism	↓ (Level 3) (Lou et al., 2024); enriched (LDA 3–4) in the case group (Marsiglia et al., 2023)	–
Fructose and mannose metabolism	–	Enriched (LDA ≥ 2.0) (Cao et al., 2025); highly enriched in both groups (Xiang et al., 2020)
Antigen processing and presentation	↑ (Level 3) (Ma et al., 2024)	↓ (Level 3) (Xu et al., 2021)
Lipoic acid metabolism	↑ (He et al., 2024)	–
Butanoate metabolism	Enriched (LDA 3–4) (Marsiglia et al., 2023)	–
Cysteine and methionine metabolism	–	Enriched (LDA 3–4) (Marsiglia et al., 2023)
Phenazine biosynthesis	↓ ; ↑ (level 3) (Wei et al., 2023; Ma et al., 2024)	–
Bifidobacterium shunt	↓ (Wei et al., 2023)	–

↑/↓, significantly increased/enriched or decreased in the respective group as reported. Enriched (LDA ≥ *x*): reported as significantly enriched via LEfSe analysis with the indicated LDA score. Level 2/3: KEGG pathway hierarchy level. Consistent marker: Pathway alteration reported in ≥2 studies with the same direction. Inconsistent/contradictory: findings differ across studies (noted in the table).

### 3.5 Correlations with clinical indicators

Twelve studies investigated the relationship between specific gut microbial genera and various clinical parameters in IS patients. The majority of reported correlations were derived from single studies, highlighting the preliminary and exploratory nature of this evidence. Among the most frequently examined genera, *Faecalibacterium* was commonly associated with favorable outcomes, showing negative correlations with stroke severity scores (NIHSS in Luo 2024 (Luo et al., 2024); mRS in Luo 2024 (Luo et al., 2024)) and inflammatory markers (NLR, MLR, IL-6 in Luo 2024 (Luo et al., 2024)) and a positive correlation with cognitive function (MoCA in Huang 2021 (Huang et al., 2021)). Conversely, *Lactobacillus* exhibited a pattern of association with worse clinical status, demonstrating positive correlations with NIHSS score (Li 2020 (Li et al., 2020)), homocysteine (Yu 2024) (Yu et al., 2024), and hypersensitive C-reactive protein (Ma 2024) (Ma et al., 2024), alongside a negative correlation with cognitive function (MoCA in Huang 2021 (Huang et al., 2021)). *Streptococcus* abundance was positively correlated with various metabolic and inflammatory indices, including serum lipids (creatinine, lipoprotein; Huang 2019 (Huang et al., 2019)), blood glucose and albumin, triglyceride levels (Marsiglia 2023) (Marsiglia et al., 2023), and fecal short-chain fatty acid levels (Marsiglia 2023) (Marsiglia et al., 2023). *Blautia* was the only genus where a specific correlation (negative correlation with NIHSS score) was reported by two independent studies (He 2024; Bao 2024) (Bao et al., 2024; He et al., 2024). A wide array of other significant correlations were reported between individual microbial genera and diverse clinical metrics, including laboratory parameters (e.g., lipids, liver enzymes, TMAO), imaging findings (infarct volume, plaque area), and functional scores (e.g., MoCA, mRS); however, each of these was reported by only a single study (**Table 6**).

**Table 6:** Correlation between characteristic gut microbiota and clinical indicators of ischemic stroke.

Microbial genus	Clinical parameter	Direction of association	Reporting studies (*n*)	Key references (first author, year)
*Faecalibacterium*	NIHSS score	Negative	1	(Luo et al., 2024)
	Modified Rankin scale (mRS)	Negative	1	(Luo et al., 2024)
	Triglycerides	Negative	1	(Marsiglia et al., 2023)
	Montreal Cognitive Assessment (MoCA)	Positive	1	(Huang et al., 2021)
	Height	Positive	1	(Li et al., 2020)
	NLR	Negative	1	(Luo et al., 2024)
	MLR	Negative	1	(Luo et al., 2024)
	IL-6	Negative	1	(Luo et al., 2024)
	PNR	Positive	1	(Luo et al., 2024)
*Lactobacillus*	NIHSS score	Positive	1	(Li et al., 2020)
	Homocysteine	Positive	1	(Yu et al., 2024)
	High-density lipoprotein cholesterol	Negative	1	(Yu et al., 2024)
	Hypersensitive C-reactive protein	Positive	1	(Ma et al., 2024)
	Cognitive Function (MoCA)	Negative	1	(Huang et al., 2021)
*Streptococcus*	High-density lipoprotein cholesterol	Negative	1	(Yu et al., 2024)
	Acetic	Positive	1	(Marsiglia et al., 2023)
	Propanoic	Positive	1	(Marsiglia et al., 2023)
	Butanoic acid	Positive	1	(Marsiglia et al., 2023)
	Glucose and albumin	Positive	1	(Marsiglia et al., 2023)
	Creatinine	Positive	1	(Huang et al., 2019)
	Lipoprotein	Positive	1	(Huang et al., 2019)
	Triglycerides	Positive	1	(Marsiglia et al., 2023)
Blautia	NIHSS score	Negative	2	(Bao et al., 2024; He et al., 2024)
	Hypersensitive C-reactive protein	Negative	1	(Ma et al., 2024)
	Discharge modified Rankin Scale (mRS)	Negative	1	(He et al., 2024)
	Blood cell count	Negative	1	(Huang et al., 2019)
	Acetic	Positive	1	(Marsiglia et al., 2023)
	Butanoic acid	Positive	1	(Marsiglia et al., 2023)
*Parabacteroides*	Infarct Volume	Positive	1	(Xu et al., 2021)
	Carotid artery plaque area	Positive	1	(Xu et al., 2021)
	Creatine kinase	Positive	1	(Xu et al., 2021)
	Total cholesterol	Positive	1	(Xu et al., 2021)
	High-density lipoprotein cholesterol	Negative	1	(Xu et al., 2021)
	Montreal cognitive assessment (MoCA)	Negative	1	(Huang et al., 2021)
	Diabetes	Positive	1	(Huang et al., 2021)
	Zonulin	Negative	1	(Marsiglia et al., 2023)
*Bifidobacterium*	Hypertension	Negative	1	(Ma et al., 2024)
	NIHSS	Positive	1	(He et al., 2024)
	Brain infarction volume	Positive	1	(He et al., 2024)
	Triglyceride	Positive	1	(He et al., 2024)
	Homocysteine (HCY)	Positive	1	(He et al., 2024)
	Discharge modified Rankin scale (mRS)	Positive	1	(He et al., 2024)
*Veillonella*	Mean arterial pressure	Positive	1	(Ma et al., 2024)
	NIHSS	Negative	1	(Ma et al., 2024)
*Dialister*	Hypersensitive C-reactive protein	Negative	1	(Ma et al., 2024)
	Uric acid	Positive	1	(Li et al., 2019)
*Lachnoclostridium*	Hypersensitive C-reactive protein	Negative	1	(Ma et al., 2024)
	Age	Negative	1	(Huang et al., 2021)
*Butyricicoccus*	Hypersensitive C-reactive protein	Negative	1	(Ma et al., 2024)
*Desulfovibrio*	Hypersensitive C-reactive protein	Positive	1	(Ma et al., 2024)
	Zonulin	Negative	1	(Marsiglia et al., 2023)
*Coprococcus*	Modified Rankin Scale (mRS)	Positive	1	(Ma et al., 2024)
	Glucose and albumin	Positive	1	(Marsiglia et al., 2023)
*Lactococcus*	Modified Rankin Scale (mRS)	Positive	1	(Ma et al., 2024)
*Prevotella*	NIHSS	Negative	1	(Ma et al., 2024)
	AST	Positive	1	(Marsiglia et al., 2023)
	Bilirubin	Positive	1	(Marsiglia et al., 2023)
	ALT	Negative	1	(Marsiglia et al., 2023)
*Akkermansia*	NIHSS	Positive	1	(Ma et al., 2024)
	Triglycerides	Positive	1	(Marsiglia et al., 2023)
	AST	Positive	1	(Marsiglia et al., 2023)
*Faecalibacterium prausnitzii*	NIHSS	Negative	1	(Luo et al., 2024)
	Modified Rankin scale (mRS)	Negative	1	(Luo et al., 2024)
	NLR	Negative	1	(Luo et al., 2024)
	MLR	Negative	1	(Luo et al., 2024)
	IL-6	Negative	1	(Luo et al., 2024)
	PNR	Positive	1	(Luo et al., 2024)
*Enterococcus*	Montreal Cognitive Assessment (MoCA)	Negative	1	(Huang et al., 2021)
*Ruminococcus*	TMAO	Positive	1	(Xu et al., 2021)
	High-density lipoprotein	Positive	1	(Li et al., 2019)
	Acetic acid	Positive	1	(Marsiglia et al., 2023)
	ALT	Negative	1	(Marsiglia et al., 2023)
	Glucose of blood	Negative	1	(Li et al., 2019)
*Dorea*	Systolic pressure	Positive	1	(Li et al., 2020)
*Alistipes*	Carotid artery plaque area	Positive	1	(Xu et al., 2021)
	Creatine kinase	Positive	1	(Xu et al., 2021)
	Total cholesterol	Positive	1	(Xu et al., 2021)
	High-density lipoprotein cholesterol	Negative	1	(Xu et al., 2021)
	TMAO	Positive	1	(Xu et al., 2021)
	Hypertension	Positive	1	(Ma et al., 2024)
	Glucose of blood	Negative	1	(Li et al., 2019)
	GGT	Positive	1	(Marsiglia et al., 2023)
	Glucose	Positive	1	(Marsiglia et al., 2023)
*Anaerotruncus*	TMAO	Positive	1	(Xu et al., 2021)
*Odoribacter*	TMAO	Positive	1	(Xu et al., 2021)
*Roseburia*	Montreal Cognitive Assessment (MoCA)	Positive	1	(Huang et al., 2021)
	Age	Negative	1	(Huang et al., 2021)
*Anaerostipes*	Montreal cognitive assessment (MoCA)	Positive	1	(Huang et al., 2021)
	Bilirubin	Positive	1	(Marsiglia et al., 2023)
	Diabetes	Positive	1	(Huang et al., 2021)
*Agathobacter*	Montreal cognitive assessment (MoCA)	Positive	1	(Huang et al., 2021)
*Bacteroides*	Diabetes	Positive	1	(Huang et al., 2021)
	Low-density lipoprotein	Positive	1	(Li et al., 2019)
	MI_TOT	Negative	1	(Marsiglia et al., 2023)
*Klebsiella*	NIHSS	Negative	1	(Li et al., 2020)
*Fusicatenibacter*	NIHSS	Negative	1	(Li et al., 2020)
	Age	Negative	1	(Li et al., 2020)
*Peptoclostridium*	Age	Negative	1	(Li et al., 2020)
*Haemophilus*	Systolic pressure	Negative	1	(Li et al., 2020)
*Enterobacter*	High-density lipoprotein	Negative	1	(Li et al., 2020)
	NIHSS	Positive	1	(Li et al., 2019)
	Modified Rankin scale (mRS)	Positive	1	(Li et al., 2019)
*Megamonas*	Homocysteine	Positive	1	(Li et al., 2019)
	Mean arterial pressure	Positive	1	(Ma et al., 2024)
*Fusobacterium*	Homocysteine	Positive	1	(Li et al., 2019)
*Lachnospira*	Butanoic	Positive	1	(Marsiglia et al., 2023)
	Propanoic acid	Positive	1	(Marsiglia et al., 2023)
*Catenibacterium*	Butanoic acid	Positive	1	(Marsiglia et al., 2023)
*Methanobrevibacter*	Acetic	Positive	1	(Marsiglia et al., 2023)
	Propanoic acid	Positive	1	(Marsiglia et al., 2023)
*Succinivibri*	Zonulin	Negative	1	(Marsiglia et al., 2023)
*Clostridium*	MI_TOT	Negative	1	(Marsiglia et al., 2023)
*Succinivibrio*	AST	Positive	1	(Marsiglia et al., 2023)
*Butyricimonas*	GGT	Positive	1	(Marsiglia et al., 2023)
*Sutterella*	GGT	Positive	1	(Marsiglia et al., 2023)
*Megasphera*	Glucose	Negative	1	(Marsiglia et al., 2023)
*Uminococcus*	Cholesterol	Positive	1	(Marsiglia et al., 2023)
*Eubacterium*	Glucose	Positive	1	(Marsiglia et al., 2023)

NLR, neutrophil-to-lymphocyte ratio; MLR, monocyte-to-lymphocyte ratio; IL-6, interleukin-6; PNR, platelet-to-neutrophil ratio; AST, aspartate aminotransferase; ALT, alanine aminotransferase; TMAO, trimethylamine N-oxide; GGT, gamma-glutamyl transpeptidase; MI_TOT, Motricity Index test.

### 3.6 Risk of bias and publication bias

Quality assessment indicated generally sound methodology, with all studies excluding participants with recent antibiotic or probiotic use. The most common limitations were a lack of dietary data and incomplete adjustment for body mass index. Due to the limited number of studies available for any single quantitative comparison, formal assessment of publication bias was not deemed appropriate.

## 4 Discussion

This systematic integration of 24 studies demonstrates a consistent association between ischemic stroke and a distinct gut microbiota configuration. The lack of consistent alpha diversity changes suggests that stroke-related dysbiosis may be more qualitative (altered composition) than quantitative (loss of overall diversity), a pattern also observed in the frailty microbiome (Almeida et al., 2022). The consistent and marked separation in beta diversity strongly supports the existence of a stroke-associated microbiota state.

The microbial signature of IS is characterized by a dual pattern: enrichment of opportunistic and pro-inflammatory taxa and depletion of beneficial, SCFA-producing taxa. The expansion of Proteobacteria (including *Escherichia_Shigella*, *Klebsiella*), *Streptococcus*, and *Enterococcus* is consistent with a state of increased gut barrier permeability and systemic inflammation, as these taxa have been associated with endotoxin production (LPS) and immune activation in previous studies (Shin et al., 2015; Wang et al., 2020; Moreira De Gouveia et al., 2024). However, whether this represents a cause or consequence of the post-stroke state cannot be determined from the current cross-sectional data. This observation is consistent with the predicted enrichment of LPS biosynthesis and flagellar assembly pathways in IS patients. Conversely, the depletion of core butyrate producers like *Faecalibacterium*, *Roseburia*, *Agathobacter*, and *Eubacterium* likely results in a deficit of butyrate, a critical SCFA with anti-inflammatory, neuroprotective, and gut barrier-strengthening properties (Martin-Gallausiaux et al., 2021; Akhtar et al., 2022). A similar depletion pattern has been observed in post-stroke depression (Li et al., 2025).

The significant correlations between specific microbes and clinical severity metrics are particularly compelling. The negative association of *Faecalibacterium* (a major butyrate producer) with stroke severity (NIHSS, mRS) and its positive link to cognition suggest a potential protective role that warrants further investigation. Conversely, the positive correlations of *Lactobacillus* and *Streptococcus* with worse outcomes appear to challenge the conventional view of *Lactobacillus* as universally probiotic and suggest that under specific conditions (e.g., post-stroke inflammation), certain species may exert different functional effects, a finding that echoes observations in other inflammatory conditions (Almeida et al., 2022). In the context of heightened gut permeability and systemic inflammation following ischemic stroke, certain *Lactobacillus* species may assume an opportunistic pathobiont-like role, as has been observed in other conditions characterized by gut barrier disruption (Di Vincenzo et al., 2024).

The functional predictions, though derived indirectly from 16S rRNA gene sequence data, paint a coherent metabolic picture: an IS gut microbiome potentially geared toward heightened pathogenic potential (LPS, flagella) and xenobiotic processing, while being deficient in vital nutritional and anti-inflammatory metabolic pathways like vitamin metabolism and SCFA production. This functional shift has been hypothesized to create a pro-inflammatory milieu that could influence post-stroke outcomes (Li et al., 2026; Li et al., 2026; Zhang et al., 2026). However, whether this mechanism operates in the present cohort cannot be determined from the current data.

To the best of our knowledge, this study provides one of the most comprehensive syntheses on the topic to date, integrating taxonomic, functional, and clinical correlation data. Nevertheless, several limitations should be acknowledged. First, most studies were conducted in Chinese populations, which may limit geographical and ethnic generalizability. Second, variability in sequencing targets and bioinformatic pipelines precluded formal meta-analysis, leading us to adopt a vote-counting approach for synthesizing abundance data. This approach does not account for effect magnitude and may be influenced by studies with larger sample sizes, limitations that should be considered alongside the methodological heterogeneity when interpreting the findings. Third, most studies are cross-sectional, hindering causal inference. With fecal samples collected exclusively after stroke onset, the current evidence cannot distinguish whether dysbiosis precedes stroke or occurs as a consequence of it. Formal causal inference methods, such as Mendelian randomization, were not applicable to the available cross-sectional data. Fourth, residual confounding cannot be excluded, as dietary information and medication use (particularly proton pump inhibitors, metformin, and statins) were incompletely documented, and comorbidities were variably reported across studies.

These findings highlight several directions for future research. To establish causality and generalizability, large-scale, multi-ethnic, longitudinal cohort studies are needed that combine standardized metagenomic sequencing with metabolomic profiling to capture direct functional data, rather than relying on 16S rRNA-based predictions. In parallel, mechanistic animal studies should test whether the key taxa identified here—such as the depleted butyrate producer *Faecalibacterium* or the enriched *Streptococcus*—play a causal role in stroke severity.

From a translational perspective, the consistent correlations between specific microbial genera and clinical outcomes support the investigation of gut microbiota-derived prognostic biomarkers, such as the *Faecalibacterium*/*Streptococcus* ratio (Safarchi et al., 2025; Gao et al., 2026). Therapeutic strategies aimed at restoring eubiosis, including targeted probiotics (e.g., butyrate-producing strains), prebiotic fiber, or fecal microbiota transplantation, warrant rigorous clinical testing as adjuncts to post-stroke management (Fang et al., 2023; Ren et al., 2025; Li et al., 2026).

However, before these strategies can be translated into clinical practice, several barriers must be addressed. Large-scale, prospective studies with standardized, multi-time-point sampling are needed to control for important confounders, including diet, medications (particularly anticoagulants and statins), and post-stroke infections. Additionally, standardized protocols for sample collection, sequencing, and data analysis are required to enable cross-study comparisons and meta-analyses.

## 5 Conclusion

This evidence synthesis establishes that ischemic stroke is reproducibly associated with a distinct gut dysbiosis pattern, featuring an increase in pro-inflammatory and pro-pathogenic bacteria and a decrease in beneficial, SCFA-producing commensals. These alterations are linked to predicted detrimental shifts in microbial community function and correlate with clinical stroke severity. However, whether this dysbiosis is a cause, consequence, or exacerbating factor of IS remains to be determined. The gut microbiota represents a promising frontier for discovering novel biomarkers and developing adjunctive therapeutic strategies to improve outcomes in ischemic stroke.

## Data Availability

Data described in the article, code book, and analytic code will not be made available because approval has not been granted by participants. Requests to access the datasets should be directed to Liangnan Zeng.
